# Health Tract, No. 124

**Published:** 1863-01

**Authors:** 


### HEALTH TRACT NO. 124.

---

### BALDNESS.

Each hair generally has one bulb or root by which it is nourished; when this root is destroyed by sickness, violence, or age, the hair can never grow again; this is the case when the scalp is shiny or glistening.

When the scalp is fuzzy, like the down of a very young bird, it is from debility of the hair-bulbs, occasioned by severe or protracted diseases; in this case, the hair grows with increasing profusion as the health recovers. Whatever hair-wash or oil happens to be applied at this conjuncture, gets the credit of a hair restorative; hence the great number of these articles, not one of the whole number being a whit more efficacious than the sprinkling of a thimbleful of ashes on the poll, except so far as they have a tendency to keep the scalp clean, which common soap-suds will abundantly do; or except they have the effect to stimulate the scalp, and promote a more vigorous circulation of the blood; but it is not possible for any oil or grease ever to do this. To make hair grow on a shining scalp is utterly impossible. But the growth of hair may be promoted on a fuzzy scalp, because in that case the root is not dead, but lacks vigor, lacks nutriment, and new vigor can be imparted, and additional nutriment bestowed by whatever gives activity to the circulation of the blood about the roots of the hair, and what the following application fails to do in this direction, all others will, simply because it is the most certain, the most powerful and safe hair stimulant known: Half an ounce of vinegar of cantharides, one ounce of cologne-water, one ounce of rose-water; to be rubbed in with a tooth-brush gently and patiently, until the part is thoroughly wetted and smarts a little; to be repeated night and morning; if too powerful, dilute with water, or use less. Age brings incurable baldness, sooner or later, to almost all; but the great object of this article is to procrastinate incurable baldness, and to prevent the premature loss or thinning of the hair: first, by avoiding the causes; second, by proper attention to promoting the growth of the hair.

The ancient Romans seldom wore any thing on the head, and a case of baldness was a rare thing.

Baldness is very infrequent among the Indians; their heads are habitually uncovered.

Baldness among women is very much rarer than among men. Women's baldness is about the temples, that of man on the top of the head. It may be then inferred that one cause of baldness is keeping the head covered and heated, thus excessively stimulating the hair-glands by an unnatural warmth, and prematurely exhausting their power, and also by preventing the evaporation and escape of that effete matter, the continued presence of which is always death, in whatever part of the system it may occur. This is effectually done by the large quantities of grease and oil which our women plaster on the sides of the head and temples, the hair, dust, and oil, making a coating over the temples almost as impervious as India-rubber, thus choking up the roots or glands and preventing the proper blood circulation; for it is the blood which carries nutriment to the hair.

The top of the head is most profusely supplied with blood-vessels, yet men grow bald there first, by keeping the head too warm; also, and chiefly, by the prevalent fashion for generations past, of wearing hard fur and silk hats, which by their pressure all around the head, forcibly detain the blood from the top of the head; there is seldom baldness below where the hat touches the head. None of the writer's playmates are known to be bald at ages from forty to sixty-five; it was the universal custom among them as boys, to wear loose woolen hats, answering to the felt hats now coming into fashion. To prevent thin hair and premature baldness, first, keep a clean scalp; second, never wear the hair on a strain, or against the direction of its growth; third, never apply any thing to it but soap-suds or pure water; fourth, wear loose-fitting, soft hats; fifth, let men and children always wear the hair very short, and both men and women should brush the hair a great deal, using only a coarse comb, which should touch the scalp only in the slightest manner possible.

